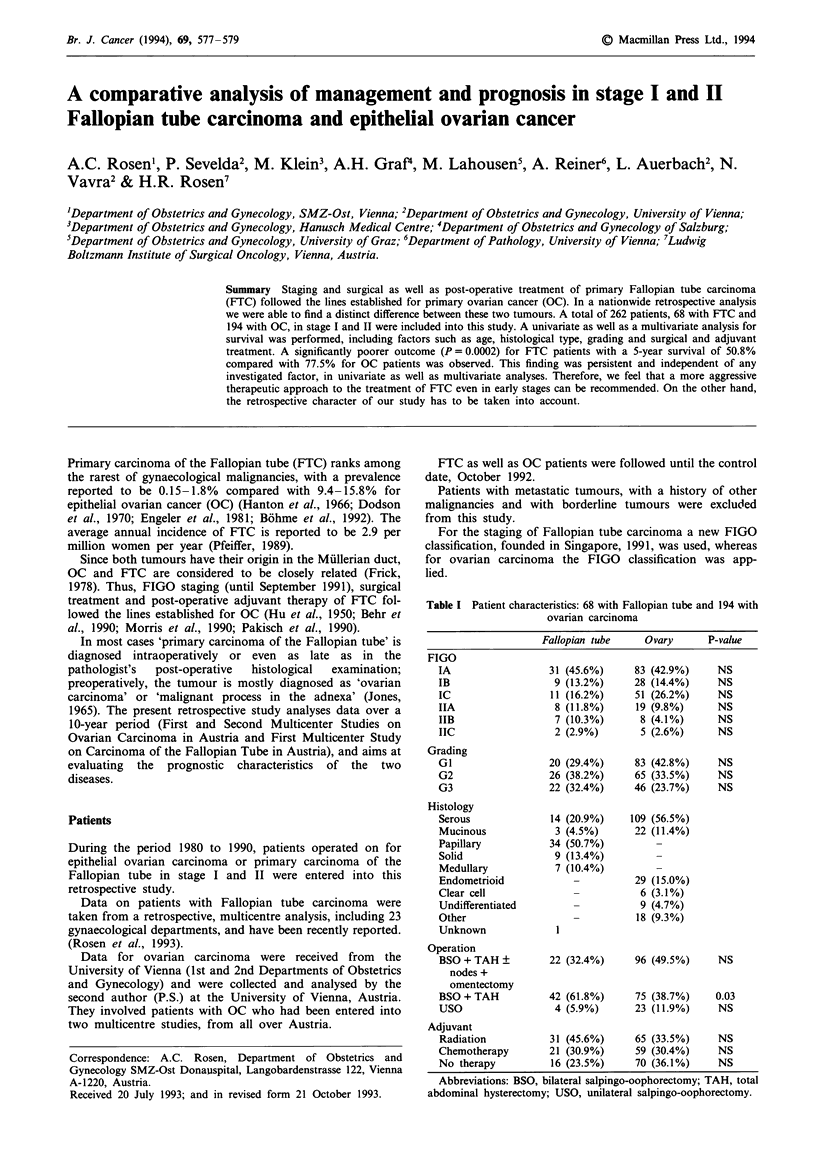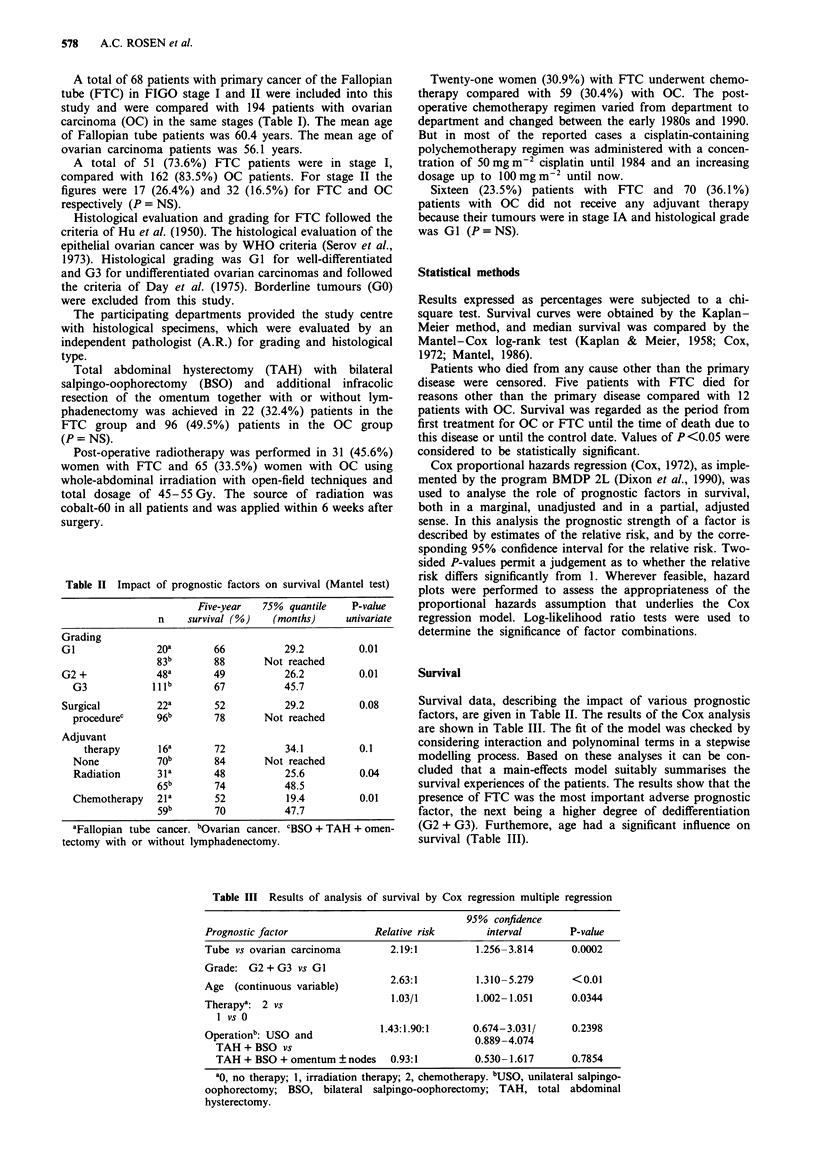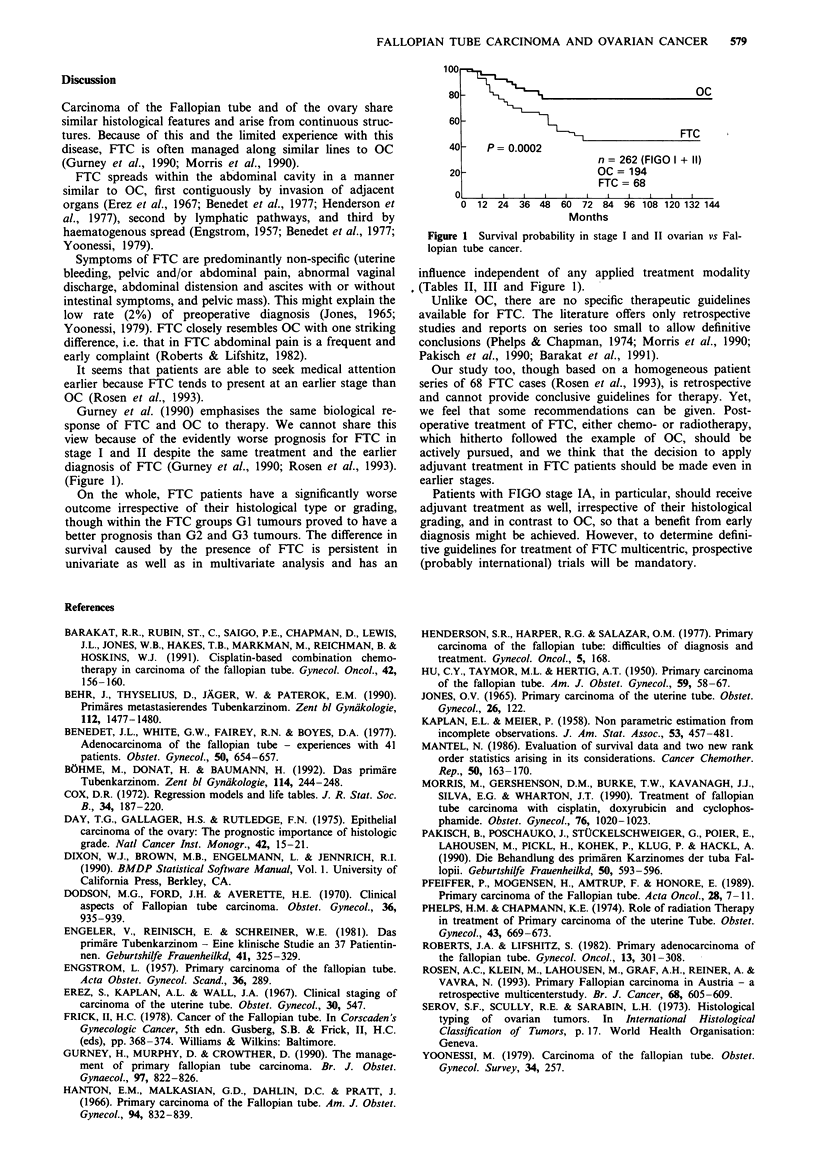# A comparative analysis of management and prognosis in stage I and II fallopian tube carcinoma and epithelial ovarian cancer.

**DOI:** 10.1038/bjc.1994.105

**Published:** 1994-03

**Authors:** A. C. Rosen, P. Sevelda, M. Klein, A. H. Graf, M. Lahousen, A. Reiner, L. Auerbach, N. Vavra, H. R. Rosen

**Affiliations:** Department of Obstetrics and Gynecology, SMZ-Ost, Vienna, Austria.

## Abstract

Staging and surgical as well as post-operative treatment of primary Fallopian tube carcinoma (FTC) followed the lines established for primary ovarian cancer (OC). In a nationwide retrospective analysis we were able to find a distinct difference between these two tumours. A total of 262 patients, 68 with FTC and 194 with OC, in stage I and II were included into this study. A univariate as well as a multivariate analysis for survival was performed, including factors such as age, histological type, grading and surgical and adjuvant treatment. A significantly poorer outcome (P = 0.0002) for FTC patients with a 5-year survival of 50.8% compared with 77.5% for OC patients was observed. This finding was persistent and independent of any investigated factor, in univariate as well as multivariate analyses. Therefore, we feel that a more aggressive therapeutic approach to the treatment of FTC even in early stages can be recommended. On the other hand, the retrospective character of our study has to be taken into account.


Br. J. Cancer (1994), 69, 577–579 © Macmillan Press Ltd., 1994

### A comparative analysis of management and prognosis in stage I and II Fallopian tube carcinoma and epithelial ovarian cancer

A.C. Rosen[1], P. Sevelda[2], M. Klein[3], A.H. Graf[4], M. Lahousen[5], A. Reiner[6], L. Auerbach[2], N. Vavra[2] & H.R. Rosen[7]

[1]*Department of Obstetrics and Gynecology, SMZ-Ost, Vienna;* [2]*Department of Obstetrics and Gynecology, University of Vienna;* [3]*Department of Obstetrics and Gynecology, Hanusch Medical Centre;* [4]*Department of Obstetrics and Gynecology of Salzburg;* [5]*Department of Obstetrics and Gynecology, University of Graz;* [6]*Department of Pathology, University of Vienna;* [7]*Ludwig Boltzmann Institute of Surgical Oncology, Vienna, Austria.*

**Summary** Staging and surgical as well as post-operative treatment of primary Fallopian tube carcinoma (FTC) followed the lines established for primary ovarian cancer (OC). In a nationwide retrospective analysis we were able to find a distinct difference between these two tumours. A total of 262 patients, 68 with FTC and 194 with OC, in stage I and II were included into this study. A univariate as well as a multivariate analysis for survival was performed, including factors such as age, histological type, grading and surgical and adjuvant treatment. A significantly poorer outcome ($P = 0.0002$) for FTC patients with a 5-year survival of 50.8% compared with 77.5% for OC patients was observed. This finding was persistent and independent of any investigated factor, in univariate as well as multivariate analyses. Therefore, we feel that a more aggressive therapeutic approach to the treatment of FTC even in early stages can be recommended. On the other hand, the retrospective character of our study has to be taken into account.

Primary carcinoma of the Fallopian tube (FTC) ranks among the rarest of gynaecological malignancies, with a prevalence reported to be 0.15–1.8% compared with 9.4–15.8% for epithelial ovarian cancer (OC) (Hanton *et al.*, 1966; Dodson *et al.*, 1970; Engeler *et al.*, 1981; Böhme *et al.*, 1992). The average annual incidence of FTC is reported to be 2.9 per million women per year (Pfeiffer, 1989).

Since both tumours have their origin in the Müllerian duct, OC and FTC are considered to be closely related (Frick, 1978). Thus, FIGO staging (until September 1991), surgical treatment and post-operative adjuvant therapy of FTC followed the lines established for OC (Hu *et al.*, 1950; Behr *et al.*, 1990; Morris *et al.*, 1990; Pakisch *et al.*, 1990).

In most cases 'primary carcinoma of the Fallopian tube' is diagnosed intraoperatively or even as late as in the pathologist's post-operative histological examination; preoperatively, the tumour is mostly diagnosed as 'ovarian carcinoma' or 'malignant process in the adnexa' (Jones, 1965). The present retrospective study analyses data over a 10-year period (First and Second Multicenter Studies on Ovarian Carcinoma in Austria and First Multicenter Study on Carcinoma of the Fallopian Tube in Austria), and aims at evaluating the prognostic characteristics of the two diseases.

### Patients

During the period 1980 to 1990, patients operated on for epithelial ovarian carcinoma or primary carcinoma of the Fallopian tube in stage I and II were entered into this retrospective study.

Data on patients with Fallopian tube carcinoma were taken from a retrospective, multicentre analysis, including 23 gynaecological departments, and have been recently reported. (Rosen *et al.*, 1993).

Data for ovarian carcinoma were received from the University of Vienna (1st and 2nd Departments of Obstetrics and Gynecology) and were collected and analysed by the second author (P.S.) at the University of Vienna, Austria. They involved patients with OC who had been entered into two multicentre studies, from all over Austria.

Correspondence: A.C. Rosen, Department of Obstetrics and Gynecology SMZ-Ost Donauspital, Langobardenstrasse 122, Vienna A-1220, Austria.
Received 20 July 1993; and in revised form 21 October 1993.

FTC as well as OC patients were followed until the control date, October 1992.

Patients with metastatic tumours, with a history of other malignancies and with borderline tumours were excluded from this study.

For the staging of Fallopian tube carcinoma a new FIGO classification, founded in Singapore, 1991, was used, whereas for ovarian carcinoma the FIGO classification was applied.

**Table I** Patient characteristics: 68 with Fallopian tube and 194 with ovarian carcinoma

| | *Fallopian tube* | *Ovary* | P-*value* |
|---|---|---|---|
| FIGO | | | |
| IA | 31 (45.6%) | 83 (42.9%) | NS |
| IB | 9 (13.2%) | 28 (14.4%) | NS |
| IC | 11 (16.2%) | 51 (26.2%) | NS |
| IIA | 8 (11.8%) | 19 (9.8%) | NS |
| IIB | 7 (10.3%) | 8 (4.1%) | NS |
| IIC | 2 (2.9%) | 5 (2.6%) | NS |
| Grading | | | |
| G1 | 20 (29.4%) | 83 (42.8%) | NS |
| G2 | 26 (38.2%) | 65 (33.5%) | NS |
| G3 | 22 (32.4%) | 46 (23.7%) | NS |
| Histology | | | |
| Serous | 14 (20.9%) | 109 (56.5%) | |
| Mucinous | 3 (4.5%) | 22 (11.4%) | |
| Papillary | 34 (50.7%) | – | |
| Solid | 9 (13.4%) | – | |
| Medullary | 7 (10.4%) | – | |
| Endometrioid | – | 29 (15.0%) | |
| Clear cell | – | 6 (3.1%) | |
| Undifferentiated | – | 9 (4.7%) | |
| Other | – | 18 (9.3%) | |
| Unknown | 1 | | |
| Operation | | | |
| BSO + TAH ± nodes + omentectomy | 22 (32.4%) | 96 (49.5%) | NS |
| BSO + TAH | 42 (61.8%) | 75 (38.7%) | 0.03 |
| USO | 4 (5.9%) | 23 (11.9%) | NS |
| Adjuvant | | | |
| Radiation | 31 (45.6%) | 65 (33.5%) | NS |
| Chemotherapy | 21 (30.9%) | 59 (30.4%) | NS |
| No therapy | 16 (23.5%) | 70 (36.1%) | NS |

Abbreviations: BSO, bilateral salpingo-oophorectomy; TAH, total abdominal hysterectomy; USO, unilateral salpingo-oophorectomy.

A total of 68 patients with primary cancer of the Fallopian tube (FTC) in FIGO stage I and II were included into this study and were compared with 194 patients with ovarian carcinoma (OC) in the same stages (Table I). The mean age of Fallopian tube patients was 60.4 years. The mean age of ovarian carcinoma patients was 56.1 years.

A total of 51 (73.6%) FTC patients were in stage I, compared with 162 (83.5%) OC patients. For stage II the figures were 17 (26.4%) and 32 (16.5%) for FTC and OC respectively ($P$ = NS).

Histological evaluation and grading for FTC followed the criteria of Hu *et al.* (1950). The histological evaluation of the epithelial ovarian cancer was by WHO criteria (Serov *et al.*, 1973). Histological grading was G1 for well-differentiated and G3 for undifferentiated ovarian carcinomas and followed the criteria of Day *et al.* (1975). Borderline tumours (G0) were excluded from this study.

The participating departments provided the study centre with histological specimens, which were evaluated by an independent pathologist (A.R.) for grading and histological type.

Total abdominal hysterectomy (TAH) with bilateral salpingo-oophorectomy (BSO) and additional infracolic resection of the omentum together with or without lymphadenectomy was achieved in 22 (32.4%) patients in the FTC group and 96 (49.5%) patients in the OC group ($P$ = NS).

Post-operative radiotherapy was performed in 31 (45.6%) women with FTC and 65 (33.5%) women with OC using whole-abdominal irradiation with open-field techniques and total dosage of 45–55 Gy. The source of radiation was cobalt-60 in all patients and was applied within 6 weeks after surgery.

**Table II** Impact of prognostic factors on survival (Mantel test)

| | n | *Five-year survival (%)* | *75% quantile (months)* | P-*value univariate* |
|---|---|---|---|---|
| Grading | | | | |
| G1 | 20[a] | 66 | 29.2 | 0.01 |
| | 83[b] | 88 | Not reached | |
| G2 + | 48[a] | 49 | 26.2 | 0.01 |
| G3 | 111[b] | 67 | 45.7 | |
| Surgical | 22[a] | 52 | 29.2 | 0.08 |
| procedure[c] | 96[b] | 78 | Not reached | |
| Adjuvant | | | | |
| therapy | 16[a] | 72 | 34.1 | 0.1 |
| None | 70[b] | 84 | Not reached | |
| Radiation | 31[a] | 48 | 25.6 | 0.04 |
| | 65[b] | 74 | 48.5 | |
| Chemotherapy | 21[a] | 52 | 19.4 | 0.01 |
| | 59[b] | 70 | 47.7 | |

[a]Fallopian tube cancer. [b]Ovarian cancer. [c]BSO + TAH + omentectomy with or without lymphadenectomy.

Twenty-one women (30.9%) with FTC underwent chemotherapy compared with 59 (30.4%) with OC. The post-operative chemotherapy regimen varied from department to department and changed between the early 1980s and 1990. But in most of the reported cases a cisplatin-containing polychemotherapy regimen was administered with a concentration of 50 mg m$^{-2}$ cisplatin until 1984 and an increasing dosage up to 100 mg m$^{-2}$ until now.

Sixteen (23.5%) patients with FTC and 70 (36.1%) patients with OC did not receive any adjuvant therapy because their tumours were in stage IA and histological grade was G1 ($P$ = NS).

### Statistical methods

Results expressed as percentages were subjected to a chi-square test. Survival curves were obtained by the Kaplan–Meier method, and median survival was compared by the Mantel–Cox log-rank test (Kaplan & Meier, 1958; Cox, 1972; Mantel, 1986).

Patients who died from any cause other than the primary disease were censored. Five patients with FTC died for reasons other than the primary disease compared with 12 patients with OC. Survival was regarded as the period from first treatment for OC or FTC until the time of death due to this disease or until the control date. Values of $P < 0.05$ were considered to be statistically significant.

Cox proportional hazards regression (Cox, 1972), as implemented by the program BMDP 2L (Dixon *et al.*, 1990), was used to analyse the role of prognostic factors in survival, both in a marginal, unadjusted and in a partial, adjusted sense. In this analysis the prognostic strength of a factor is described by estimates of the relative risk, and by the corresponding 95% confidence interval for the relative risk. Two-sided $P$-values permit a judgement as to whether the relative risk differs significantly from 1. Wherever feasible, hazard plots were performed to assess the appropriateness of the proportional hazards assumption that underlies the Cox regression model. Log-likelihood ratio tests were used to determine the significance of factor combinations.

### Survival

Survival data, describing the impact of various prognostic factors, are given in Table II. The results of the Cox analysis are shown in Table III. The fit of the model was checked by considering interaction and polynominal terms in a stepwise modelling process. Based on these analyses it can be concluded that a main-effects model suitably summarises the survival experiences of the patients. The results show that the presence of FTC was the most important adverse prognostic factor, the next being a higher degree of dedifferentiation (G2 + G3). Furthemore, age had a significant influence on survival (Table III).

**Table III** Results of analysis of survival by Cox regression multiple regression

| *Prognostic factor* | *Relative risk* | *95% confidence interval* | P-*value* |
|---|---|---|---|
| Tube *vs* ovarian carcinoma | 2.19:1 | 1.256–3.814 | 0.0002 |
| Grade: G2 + G3 *vs* G1 | 2.63:1 | 1.310–5.279 | <0.01 |
| Age (continuous variable) | 1.03/1 | 1.002–1.051 | 0.0344 |
| Therapy[a]: 2 *vs* 1 *vs* 0 | 1.43:1.90:1 | 0.674–3.031/ 0.889–4.074 | 0.2398 |
| Operation[b]: USO and TAH + BSO *vs* TAH + BSO + omentum ± nodes | 0.93:1 | 0.530–1.617 | 0.7854 |

[a]0, no therapy; 1, irradiation therapy; 2, chemotherapy. [b]USO, unilateral salpingo-oophorectomy; BSO, bilateral salpingo-oophorectomy; TAH, total abdominal hysterectomy.

### Discussion

Carcinoma of the Fallopian tube and of the ovary share similar histological features and arise from continuous structures. Because of this and the limited experience with this disease, FTC is often managed along similar lines to OC (Gurney *et al.*, 1990; Morris *et al.*, 1990).

FTC spreads within the abdominal cavity in a manner similar to OC, first contiguously by invasion of adjacent organs (Erez *et al.*, 1967; Benedet *et al.*, 1977; Henderson *et al.*, 1977), second by lymphatic pathways, and third by haematogenous spread (Engstrom, 1957; Benedet *et al.*, 1977; Yoonessi, 1979).

Symptoms of FTC are predominantly non-specific (uterine bleeding, pelvic and/or abdominal pain, abnormal vaginal discharge, abdominal distension and ascites with or without intestinal symptoms, and pelvic mass). This might explain the low rate (2%) of preoperative diagnosis (Jones, 1965; Yoonessi, 1979). FTC closely resembles OC with one striking difference, i.e. that in FTC abdominal pain is a frequent and early complaint (Roberts & Lifshitz, 1982).

It seems that patients are able to seek medical attention earlier because FTC tends to present at an earlier stage than OC (Rosen *et al.*, 1993).

Gurney *et al.* (1990) emphasises the same biological response of FTC and OC to therapy. We cannot share this view because of the evidently worse prognosis for FTC in stage I and II despite the same treatment and the earlier diagnosis of FTC (Gurney *et al.*, 1990; Rosen *et al.*, 1993). (Figure 1).

On the whole, FTC patients have a significantly worse outcome irrespective of their histological type or grading, though within the FTC groups G1 tumours proved to have a better prognosis than G2 and G3 tumours. The difference in survival caused by the presence of FTC is persistent in univariate as well as in multivariate analysis and has an influence independent of any applied treatment modality (Tables II, III and Figure 1).

**Figure 1** Survival probability in stage I and II ovarian *vs* Fallopian tube cancer.

Unlike OC, there are no specific therapeutic guidelines available for FTC. The literature offers only retrospective studies and reports on series too small to allow definitive conclusions (Phelps & Chapman, 1974; Morris *et al.*, 1990; Pakisch *et al.*, 1990; Barakat *et al.*, 1991).

Our study too, though based on a homogeneous patient series of 68 FTC cases (Rosen *et al.*, 1993), is retrospective and cannot provide conclusive guidelines for therapy. Yet, we feel that some recommendations can be given. Postoperative treatment of FTC, either chemo- or radiotherapy, which hitherto followed the example of OC, should be actively pursued, and we think that the decision to apply adjuvant treatment in FTC patients should be made even in earlier stages.

Patients with FIGO stage IA, in particular, should receive adjuvant treatment as well, irrespective of their histological grading, and in contrast to OC, so that a benefit from early diagnosis might be achieved. However, to determine definitive guidelines for treatment of FTC multicentric, prospective (probably international) trials will be mandatory.

### References

BARAKAT, R.R., RUBIN, ST., C., SAIGO, P.E., CHAPMAN, D., LEWIS, J.L., JONES, W.B., HAKES, T.B., MARKMAN, M., REICHMAN, B. & HOSKINS, W.J. (1991). Cisplatin-based combination chemotherapy in carcinoma of the fallopian tube. *Gynecol. Oncol.*, **42**, 156-160.

BEHR, J., THYSELIUS, D., JÄGER, W. & PATEROK, E.M. (1990). Primäres metastasierendes Tubenkarzinom. *Zent bl Gynäkologie*, **112**, 1477-1480.

BENEDET, J.L., WHITE, G.W., FAIREY, R.N. & BOYES, D.A. (1977). Adenocarcinoma of the fallopian tube – experiences with 41 patients. *Obstet. Gynecol.*, **50**, 654-657.

BÖHME, M., DONAT, H. & BAUMANN, H. (1992). Das primäre Tubenkarzinom. *Zent bl Gynäkologie*, **114**, 244-248.

COX, D.R. (1972). Regression models and life tables. *J. R. Stat. Soc. B.*, **34**, 187-220.

DAY, T.G., GALLAGER, H.S. & RUTLEDGE, F.N. (1975). Epithelial carcinoma of the ovary: The prognostic importance of histologic grade. *Natl Cancer Inst. Monogr.*, **42**, 15-21.

DIXON, W.J., BROWN, M.B., ENGELMANN, L. & JENNRICH, R.I. (1990). *BMDP Statistical Software Manual*, Vol. 1. University of California Press, Berkley, CA.

DODSON, M.G., FORD, J.H. & AVERETTE, H.E. (1970). Clinical aspects of Fallopian tube carcinoma. *Obstet. Gynecol.*, **36**, 935-939.

ENGELER, V., REINISCH, E. & SCHREINER, W.E. (1981). Das primäre Tubenkarzinom – Eine klinische Studie an 37 Patientinnen. *Geburtshilfe Frauenheilkd*, **41**, 325-329.

ENGSTROM, L. (1957). Primary carcinoma of the fallopian tube. *Acta Obstet. Gynecol. Scand.*, **36**, 289.

EREZ, S., KAPLAN, A.L. & WALL, J.A. (1967). Clinical staging of carcinoma of the uterine tube. *Obstet. Gynecol.*, **30**, 547.

FRICK, II, H.C. (1978). Cancer of the Fallopian tube. In *Corscaden's Gynecologic Cancer*, 5th edn. Gusberg, S.B. & Frick, II, H.C. (eds), pp. 368-374. Williams & Wilkins: Baltimore.

GURNEY, H., MURPHY, D. & CROWTHER, D. (1990). The management of primary fallopian tube carcinoma. *Br. J. Obstet. Gynaecol.*, **97**, 822-826.

HANTON, E.M., MALKASIAN, G.D., DAHLIN, D.C. & PRATT, J. (1966). Primary carcinoma of the Fallopian tube. *Am. J. Obstet. Gynecol.*, **94**, 832-839.

HENDERSON, S.R., HARPER, R.G. & SALAZAR, O.M. (1977). Primary carcinoma of the fallopian tube: difficulties of diagnosis and treatment. *Gynecol. Oncol.*, **5**, 168.

HU, C.Y., TAYMOR, M.L. & HERTIG, A.T. (1950). Primary carcinoma of the fallopian tube. *Am. J. Obstet. Gynecol.*, **59**, 58-67.

JONES, O.V. (1965). Primary carcinoma of the uterine tube. *Obstet. Gynecol.*, **26**, 122.

KAPLAN, E.L. & MEIER, P. (1958). Non parametric estimation from incomplete observations. *J. Am. Stat. Assoc.*, **53**, 457-481.

MANTEL, N. (1986). Evaluation of survival data and two new rank order statistics arising in its considerations. *Cancer Chemother. Rep.*, **50**, 163-170.

MORRIS, M., GERSHENSON, D.M., BURKE, T.W., KAVANAGH, J.J., SILVA, E.G. & WHARTON, J.T. (1990). Treatment of fallopian tube carcinoma with cisplatin, doxyrubicin and cyclophosphamide. *Obstet. Gynecol.*, **76**, 1020-1023.

PAKISCH, B., POSCHAUKO, J., STÜCKELSCHWEIGER, G., POIER, E., LAHOUSEN, M., PICKL, H., KOHEK, P., KLUG, P. & HACKL, A. (1990). Die Behandlung des primären Karzinomes der tuba Fallopii. *Geburtshilfe Frauenheilkd*, **50**, 593-596.

PFEIFFER, P., MOGENSEN, H., AMTRUP, F. & HONORE, E. (1989). Primary carcinoma of the Fallopian tube. *Acta Oncol.*, **28**, 7-11.

PHELPS, H.M. & CHAPMANN, K.E. (1974). Role of radiation Therapy in treatment of Primary carcinoma of the uterine Tube. *Obstet. Gynecol.*, **43**, 669-673.

ROBERTS, J.A. & LIFSHITZ, S. (1982). Primary adenocarcinoma of the fallopian tube. *Gynecol. Oncol.*, **13**, 301-308.

ROSEN, A.C., KLEIN, M., LAHOUSEN, M., GRAF, A.H., REINER, A. & VAVRA, N. (1993). Primary Fallopian carcinoma in Austria – a retrospective multicenterstudy. *Br. J. Cancer*, **68**, 605-609.

SEROV, S.F., SCULLY, R.E. & SARABIN, L.H. (1973). Histological typing of ovarian tumors. In *International Histological Classification of Tumors*, p. 17. World Health Organisation: Geneva.

YOONESSI, M. (1979). Carcinoma of the fallopian tube. *Obstet. Gynecol. Survey*, **34**, 257.